# The Significance of a Common Idiotype (1F7) on Antibodies against Human Immune Deficiency Virus Type 1 and Hepatitis C Virus

**DOI:** 10.3389/fonc.2016.00011

**Published:** 2016-02-05

**Authors:** Sybille Muller, Matthew S. Parsons, Heinz Kohler, Michael Grant

**Affiliations:** ^1^Immpheron, Inc., Lexington, KY, USA; ^2^Department of Microbiology and Immunology, The Peter Doherty Institute for Infection and Immunity, University of Melbourne, Melbourne, VIC, Australia; ^3^Department of Microbiology and Immunology, University of Kentucky, Lexington, KY, USA; ^4^Immunology and Infectious Diseases Program, Division of BioMedical Sciences, Faculty of Medicine, Memorial University of Newfoundland, St. John’s, NL, Canada

**Keywords:** idiotype, 1F7, HIV, SIV, HCV

## Abstract

In this review, we trace the concept and potential functional role of regulatory idiotypes in the immune response to human immunodeficiency virus type 1 (HIV-1), simian immunodeficiency virus, and hepatitis C virus (HCV). A major idiotype involved in these viral infections is recognized and defined by a murine monoclonal antibody (1F7). Antibodies expressing the idiotype defined by 1F7 are dominant in HIV-1 infection and are also found on many broadly neutralizing antibodies against HIV-1. This regulatory idiotypic axis offers opportunities for exploitation in vaccine development for HIV-1, HCV, and other chronic viral infections.

## Anti-Idiotypic Antibodies and Immune Regulation

Oudin and Jerne are the founders of the Idiotype Concept (1, 2). They “discovered” that one antibody could recognize another antibody as an individual and unique member of the immune system. The uniqueness of an antibody lies in the variable region of heavy and light chains and has been named idiotype. Essential insight into the Idiotypic network has emerged from studies on the immune response of mice to phosphorylcholine (PC) [for review, see Ref. (3)]. Evidence that idiotype–anti-idiotype interactions may play a role in the regulation of the immune response came in part from the observation that auto-anti-idiotypic antibodies appear during an immune response to PC (4, 5). Similar evidence of auto-anti-idiotypic antibodies was later discovered in human immunodeficiency virus type 1 (HIV-1)-infected individuals who had antibodies against the 1F7 idiotype (6).

The utility of anti-idiotypic antibodies as immune regulatory molecules was initially demonstrated by Cosenza and Kohler who showed that an anti-idiotype antibody suppressed the response to PC (7, 8). Eichmann and Rajewski later suggested that anti-idiotype antibodies could be used as vaccines (9). In the following decades, anti-idiotypic antibodies have been investigated and probed as therapeutic antibodies in a variety of infectious diseases and malignancies. These anti-idiotypes were used successfully as surrogate antigens, termed Ab2beta, internal image or network antigen, thus replacing the original antigens used as therapeutic vaccines (10).

Anti-idiotypic antibodies were defined as Ab2alpha, Ab2beta, and Ab2gamma, reflecting their biological function as therapeutic antibodies. While Ab2alphas are non-internal images, which are non-biologically effective antibodies, Ab2gammas are non-internal images, which are biologically effective antibodies. Therefore, Ab2beta and Ab2gamma, being both biological effective as therapeutic antibodies, were integrated under the term network antigens (11). The term “idiotype” is used to refer to the entire collection of unique idiotopes within the variable region of a monoclonal antibody (mAb), whereas the term “idiotope” refers to a single determinant recognized within the variable region of an antibody.

Idiotypes expressed on anti-pathogen antibodies might also be utilized as disease markers (12). In the following, we will review the recent progress or lack thereof in the utilization of anti-idiotypes as therapeutic antibodies or disease markers. We will focus on HIV-1 and hepatitis C virus (HCV) infection as both HIV-1 and HCV can lead to cancer in late stages of infectious disease (13, 14).

## Expression of the 1F7 Idiotype on Antibodies against Immune Deficiency-Causing Retroviruses

Several HIV infection-related anti-idiotype studies preceded 1F7 anti-idiotype research. These previous studies demonstrated that, in contrast to 1F7, some anti-idiotypes could be used as surrogate antigen to stimulate immune responses against HIV.

Morrow et al. demonstrated that anti-idiotypic antisera against a mAb specific for a p24 gag region epitope detected a common interspecies idiotype associated with anti-HIV response (15). Kang et al. produced an anti-idiotypic mAb (3C9) related to gp120-affinity purified human anti-gp120 antibodies directed against the conserved CD4 attachment site of gp120. It was subsequently shown that immunization with anti-idiotype 3C9 elicited broadly neutralizing antibodies (BnAbs) in naive, non-HIV-infected monkeys (16).

The murine monoclonal antibody defining the 1F7 idiotype, and named 1F7, was originally raised against pooled human antibodies against HIV-1, subcloned, and further selected by ELISA against specific anti-HIV-1 antibodies captured by HIV-1 proteins gp120 and p24 (17). Pooled human immunoglobulin from non-HIV-1-infected individuals (IVIG) was used as negative control in the selection process. Further investigation revealed that human monoclonal anti-gp120, and anti-p24 antibodies, as well as anti-HIV-1 human serum antibodies, share the common idiotype/clonotype 1F7 (18).

Subsequent research identified a region (FR3–CDR3) on three human mAb directed against gp120 or p24 that bound to the murine anti-1F7 mAb, allowing production of a peptide mimicking the idiotypic region. Design of the peptide was based on the molecular recognition theory. As such, regions of inverse hydropathy between the variable sequence of the anti-1F7 and the human mAb, which are assumed to be involved in the idiotype–anti-idiotype contacts, were selected for the peptide design (19). Human anti-HIV-1 serum antibodies from a variety of HIV-1-infected individuals bound to this peptide, indicating an auto-anti-idiotypic humoral immune response to the 1F7 idiotype (6). Further evidence of a biological role for the 1F7 idiotype in HIV-1 infection was provided by studies of cellular immunity. Culture of peripheral blood mononuclear cells (PBMC) from HIV-1-infected individuals with 1F7-induced apoptosis of CD4^+^ and CD8^+^ T cells and the 1F7 mAb selectively blocked cytotoxicity of CD8^+^ T cells from HIV-1-infected individuals (20, 21). The 1F7 idiotype is also expressed on antibodies against the envelope glycoprotein of simian immunodeficiency virus (SIV)- and chimeric simian-human immunodeficiency virus (SHIV) in infected rhesus monkeys (22, 23). As such, the impact of idiotypic regulation through targeting the 1F7 idiotype was explored in the SHIV-IIIB infection model (24, 25). Suppressing expression of the 1F7 idiotype through administration of the murine 1F7 mAb to macaques chronically infected with SHIV-IIIB increased the potency and breadth of neutralizing antibodies, with an increase in neutralization titer against HIV-IIIB and cross-neutralization of HIV-MN as tested by syncytium forming microassay (25). This was interpreted as a result of relieving clonal dominance of the 1F7 idiotype in the humoral response to SHIV, such that new antibodies could arise.

The murine 1F7 mAb has been designated as an Ab2delta antibody to distinguish it from Ab2alpha, Ab2beta, and Ab2gamma anti-idiotypes (26). Recently, we found that this anti-idiotypic clone also stained local SIV gp41-antibodies emerging in naive macaques after intravenous vaccination with SIVmac239 delta Nef and vaginal challenge with wild type SIV (27). Local antibody production in the genital tract correlated with the maturation of protection against high-dose pathogenic SIV vaginal challenge (28).

## Expression of the 1F7 Idiotype on Anti-HCV Antibodies

Since 1F7 is a widely shared idiotype/clonotype, some cross-reactivity with antibodies directed to similar structures is not surprising. Parallel research revealed that the 1F7 idiotype is also expressed on antibodies against a number of different HCV proteins (29). Since approximately 20% of HCV-exposed individuals spontaneously clear HCV infection and chronic HCV infection can be cured through treatment, this finding enabled investigation of a possible relationship between expression levels of antibodies bearing the 1F7 idiotype and development or persistence of chronic infection (30). Direction of anti-pathogen immune responses along pathways with inherent limitations as to the potency of induced antibodies was previously suggested as a mechanism by which chronic pathogens may have evolved to subvert the immune response (29, 31, 32). For example, if the dominant antigens of the pathogen have some similarity to self antigens, then conserved aspects of immunological self-tolerance could limit the efficacy of responses against the pathogen (33, 34). In the context of idiotypic regulation of the immune system, we have also proposed that chronic pathogens may have evolved to stimulate immune responses along conserved idiotypic axes defining areas of high network connectivity (29, 30). Stimulating B cells with extensive internal connectivity would propagate high levels of idiotypic recognition with reciprocal stimulation of potentially irrelevant B cell clones and favor chronic oligoclonal activation over the focused selection of high-affinity antibodies with effective potency against the pathogen. Davtyan et al. compared levels of HCV core-specific antibodies expressing the 1F7 idiotype in HCV-exposed individuals who either cleared infection or progressed to chronic infection. Levels of 1F7 idiotype expression were significantly higher on anti-HCV core antibodies from HCV-exposed individuals who progressed to chronic infection than on the anti-HCV core antibodies of those who spontaneously cleared HCV infection (30). In addition, a hierarchy of 1F7 idiotype expression levels was discernable in total circulating IgG and IgM with chronic infection greater than spontaneous clearance greater than HCV non-exposed individuals (30). The concept that pathogens establishing chronic infection, despite strong humoral responses, have evolved to select antibodies along a common idiotypic axis of the immune network represented by the 1F7 idiotype may also relate to previous studies of a public idiotype termed 16/6. Elevated levels of the 16/6 idiotype occur in autoimmune disease and microbial infection, while injection of mice with antibodies bearing the 16/6 idiotype induces chronic immune activation and systemic lupus erythematosus (SLE)-like symptoms (35–38). In this case, there also appears to be a link between induction of antibodies along a common idiotypic axis demarcated by the 16/6 idiotype, immune activation, and autoreactivity. Based on these studies, the concept of pathogenic idiotypes arose, where idiotypes such as 16/6 identify antibodies that are directly autoreactive or have the capacity to disturb idiotypic regulation in ways that promote chronic activation and development of autoimmunity (39, 40). Identification of the distinct B1 B cell subset as the primary source of neonatal and natural antibodies with both a high frequency of autoreactivity and high connectivity apparently separated the B cell repertoire into subdivisions engaged in developmental regulation versus protection against pathogens. Although B1 B cells are distinguished by their developmental pathway, T cell independence and scarcity of somatic hypermutation or isotype switching, their use of the same set of germ-line antigen receptor genes as B2 B cells guarantees some level of idiotypic overlap between the two repertoires.

The fraction of CD5-expressing (B1) and CD5-negative B cells carrying the 1F7 idiotype was measured in uninfected controls and individuals with chronic HCV infection by flow cytometry. While there was expansion of 1F7-expressing B cells in both subsets in chronic HCV infection, it was clearly skewed toward the CD5-expressing subset with up to 30% of B1 B cells expressing the 1F7 Id in some HCV-infected individuals compared to <2% in uninfected controls (30). Despite this marked effect of HCV infection on expansion of B1 B cells expressing the 1F7 idiotype, the majority of circulating antibodies against HCV and HIV-1 that express the 1F7 idiotype have undergone secondary receptor modification and, therefore, appear to originate from the B2 B cell subset (17, 18, 22, 23, 29).

## Anti-HIV-1 Antibody Breadth within the 1F7 Idiotype

Engagement of both the B1 and B2 subsets in response to an infection is unusual with respect to their perceived independent roles, different interactions with T cells, level of connectivity, and frequency of autoreactivity. Chronic infection allows for the investigation of the long-term influence that selective expression of a common idiotype has on evolution of antibodies against an infectious agent. Despite demonstrations of anti-HIV-1 antibodies against different proteins and across numerous HIV-1-infected donors carrying the 1F7 idiotype; until recently, little attention was paid to the breadth of these idiotypic antibodies (17, 18). An ideal starting point for assessing the breadth of 1F7-idiotypic antibodies is to screen anti-HIV-1 BnAbs for expression of the idiotype as the process of antibody evolution within a common idiotypic space is intricately illustrated in the development of BnAbs against HIV-1. Anti-HIV-1 BnAbs are a subset of antibodies capable of neutralizing wide arrays of HIV-1 isolates, both within and across viral subtypes (41). These antibodies have great potential as prophylactics against HIV-1, as upon passive transfer or gene delivery to macaques and humanized mice they provide protection from challenges with SHIV and HIV-1, respectively (42–46). Approximately 20% of HIV-1-infected individuals develop antibodies with broadly neutralizing activity against different HIV viruses (47). The HIV-1 BnAbs are unique in that the variable regions have undergone extensive levels of somatic hypermutation not replicated using currently available vaccine formulations (48). Germ-line precursors of the HIV-1 BnAb generally bind HIV-1 envelope glycoproteins poorly, if at all (49). As such, selection of such antibodies may initially require non-HIV-1 antigens (50). Fine-tuning the anti-HIV-1 specificity of these antibodies, as well as the generation of broad neutralization capacity, would likely require additional rounds of selection and somatic hypermutation. To assess the breadth of 1F7-idiotypic antibodies, Parsons et al. screened six well-characterized HIV-1 BnAbs (i.e., 2G12, b12, VRC01, 2F5, 4E10, and Z13e1) for expression of the 1F7 idiotype *via* ELISA, and demonstrated the presence of the 1F7 idiotype on all six antibodies (51). Given that 1F7-idiotypic antibodies are observed within a majority of HIV-1-infected individuals, but BnAbs are only detected in approximately 20% of infected individuals, this observation raised questions about how BnAbs develop from antibodies within the relatively common 1F7-idiotypic node.

Previous studies demonstrated that anti-HIV-1 antibody responses are subject to a form of original antigenic sin, known as repertoire freeze *and deceptive imprinting* (32, 52, 53). Establishment of antibodies against the contemporaneous autologous virus initiates this phenomenon during acute infection. Although the virus rapidly escapes to evade the abilities of these antibodies to suppress viral replication, B-cells carrying B-cell receptors (BCR) corresponding to these antibodies sufficiently recognize the escaped virus to be selected to undergo further rounds of somatic hypermutation (or at least keep responding to subsequent antigenic challenges). The continuous reselection of these early-established B-cell responses sustains the early-induced antibody response at the expense of novel B-cell responses more specific for the contemporaneous autologous viruses of chronic infection. Evidence of this phenomenon includes maintenance of skewed κ/λ ratios throughout infection, restricted and biased variable region gene usage throughout infection and persistence of antibody clones recognizing earlier viral variants, despite escape many months prior (54–57). Given the occurrence of repertoire freeze during HIV-1 infection, Parsons et al. hypothesized that BnAbs develop from 1F7-idiotypic antibodies established during early infection through multiple rounds of selection and somatic hypermutation (51). To assess this possibility, they screened serial plasma samples from six HIV-infected donors over time points ranging from acute through chronic infection to determine if 1F7-idiotypic antibodies arise during acute infection and are sustained throughout infection. Indeed, 1F7-idiotypic antibodies were detected in plasma samples collected within the first 3 months of HIV-1 infection, and these antibodies were expanded and sustained with longer duration of infection. This observation suggests that 1F7-idiotypic antibodies engaged during acute infection and sustained throughout infection in an idiotypic repertoire freeze are driven through repeated selection and somatic hypermutation to develop into BnAbs. This putative route to BnAb development is consistent with pathways hypothesized by others and has implications for designing BnAb-inducing vaccines.

Consistent with the hypothesis that BnAbs develop through repeated selection and somatic hypermutation of 1F7-idiotypic antibodies induced early in infection, Liao et al. recently demonstrated directly that antibodies capable of neutralizing a broad range of HIV-1 isolates develop as a result of repeated selection and mutation of antibodies by constantly evolving autologous HIV-1 (58). Haynes et al. hypothesized that BnAbs might be inducible through vaccination strategies that sequentially expose vaccine recipients to HIV-1 envelope antigens selected on the basis of binding to chronologically relevant antibody isolates, derived from HIV-1-infected donors that developed BnAbs (59). This vaccination strategy might benefit from inclusion of a prime and/or boost with F(ab)_2_ of the murine monoclonal anti-idiotypic antibody used to detect the 1F7 idiotype in *in vitro* assays. As anti-HIV-1 BnAbs appear to be selected from the 1F7-idiotypic repertoire, a prime with the anti-1F7 F(ab)_2_ should focus the B-cell response of later immunizations with relevant HIV-1 envelope antigens within the relevant B-cell repertoire. Similarly, boosts with anti-1F7 F(ab)_2_ might have the benefit of increasing the frequency of the relevant B-cell responses induced by the sequential vaccination strategy.

In addition to the ability of BnAb to prevent HIV-1 infection, non-neutralizing anti-HIV-1 antibodies have also recently gained attention as potentially protective moieties. Indeed, the ability of non-neutralizing antibodies to mediate antibody-dependent cellular cytotoxicity (ADCC) against HIV-1 was linked to the partial success of the RV144 Thai vaccine trial (60–62). Similarly, the ability of antibodies to mediate ADCC has been linked to protection against infection with pathogenic SIV challenge in macaques immunized with live-attenuated SIV (63). The ADCC competency of antibodies passively transferred *via* breast milk from HIV-1-infected mothers to their children was also associated with a lower likelihood of mother-to-child transmission (64). The breadth of these non-neutralizing ADCC competent antibodies, defined as the ability of such antibodies to trigger ADCC against target cells expressing HIV-1 envelope antigens from different viral subtypes, appears important to their protective capacity. In fact, a recent assessment of the breadth of ADCC antibodies from HIV-1-infected controllers and progressors demonstrated more breadth in controllers (65). Therefore, it is likely imperative to understand the origin of HIV-1 envelope binding antibodies with broad recognition profiles. In this regard, Parsons et al. recently assessed expression of the 1F7 idiotype on broadly reactive anti-HIV-1 antibodies from individuals infected with HIV-1 subtype B that cross-reacted with HIV-1 envelopes (i.e., gp140) from subtype B, A, and AE viral isolates (66). The 1F7 idiotype was detected on antibodies binding to each HIV-1 subtype envelope, suggesting broad recognition within the anti-HIV-1 1F7-idiotypic node. Similarly, when expression of the 1F7 idiotype was assessed on subtype B envelope (i.e., gp120)-binding anti-HIV-1 antibodies derived from individuals infected with non-subtype B viruses, the idiotype was observed on antibodies from 15/19 donors. Together with data regarding 1F7-idiotypic expression on anti-HIV-1 BnAbs, the expression profile of the 1F7 idiotype on anti-HIV-1-binding antibodies with broad recognition capabilities suggests that vaccines focusing anti-HIV-1 antibody responses within the 1F7-idiotypic repertoire can achieve a substantial breadth of HIV-1 recognition.

**Figure 1:**
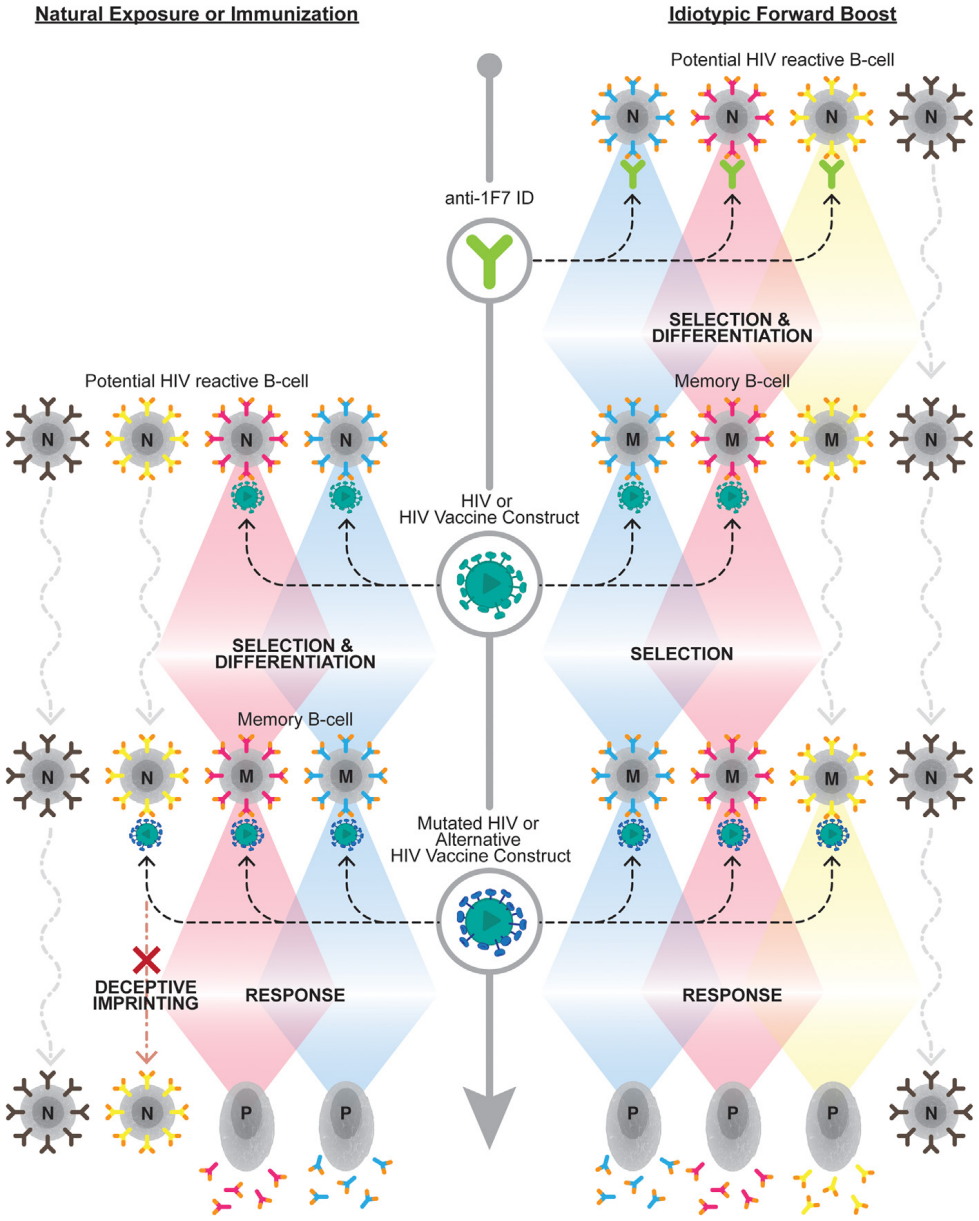
**Overcoming deceptive imprinting through forward boosting**. The left side of the diagram represents the B cell response to HIV-1 upon natural exposure to HIV-1 or immunization with an HIV-1 vaccine construct. This exposure results in a subset of naive B cell clones (*N*), which carry B cell receptors capable of binding HIV-1 antigens, being selected to undergo differentiation into memory B cells (*M*). This initial selection process induces a phenomenon known as deceptive imprinting. This phenomenon results in a reselection of previously selected B cell clones, at the expense of naive B cells carrying novel antigen-binding B cell receptor specificities, upon exposure to mutated HIV-1 or alternative HIV-1 vaccine constructs. The right side of the diagram depicts the forward-boosting strategy. This strategy predicts priming 1F7-idiotypic B cell clones (i.e., those expressing orange tipped B cell receptors), which exhibit broad HIV-1 reactivity, will establish a larger repertoire of B cell clones that can contribute to the anti-HIV-1 antibody response following future exposure or immunization. Upon future exposure to HIV-1 or an HIV-1 vaccine construct boosting of a subset of the memory B cells established from the initial anti-1F7-idiotype prime will be observed. Additional anti-1F7-idiotype primed clones should be boosted, and not impeded by deceptive imprinting, upon exposure to cognate antigens on mutated HIV-1 or alternative HIV-1 vaccine constructs. The bottom of the diagram, on both the left and right sides, demonstrates that selected B cell clones contribute to the antibody response upon becoming long-lived plasma cells (*P*).

How best to exploit the immense breadth available within the 1F7-idiotype domain remains an open question. The potential breadth of this node may be dramatically narrowed by repertoire freezes induced by HIV-1 infection itself or by vaccination with single strains. Recently, Kohler discussed that the establishment of such repertoire freezes results in “back-boosts” upon exposure to antigenically related, yet distinct, antigens, as would be the case upon challenge with a different viral strain than that utilized for vaccination (67). He further proposed utilizing the full breadth of the 1F7-idiotypic repertoire by “forward-boosting” or priming the entire 1F7 repertoire prior to exposure to any HIV-1 antigens. Future research should evaluate the ability of such a “forward-boosting” protocol to establish broadly reactive anti-HIV-1 antibodies capable of mediating potent effector functions against HIV-1 or HIV-1 infected cells (see Figure 1 depicting the forward boosting strategy).

## Summary

The concept and significance of dominant idiotypes surfaced more than 40 years ago, and the practical value of internal image antibodies or network antigens as immunogens was realized shortly afterwards. Regulatory idiotypes, the levels of which might reflect immune trajectories toward autoimmunity or immune deficiency, were proposed in the mid 1980s and continue to be studied today in autoimmunity, cancer, and infectious disease. In this review, we described how antibodies against multiple antigens of retroviruses capable of causing immune deficiency syndromes, including HIV-1, SIV, and SHIV, and also antibodies against HCV share a common idiotype designated 1F7. Expression of this idiotype is associated with a form of clonal dominance that appears to restrict development of neutralizing antibodies against contemporaneous infecting strains of HIV-1 and imposes a trajectory toward chronic infection with HCV. However, BnAbs that develop against HIV-1 do bear the 1F7 idiotype, indicating that the same idiotypic axis accommodating clonal dominance and original antigenic sin also accommodates antibody evolution toward the highly mutated progeny capable of neutralizing diverse strains of HIV-1. Learning how to exploit this axis selectively to prime for the development of BnAbs in advance of viral exposure may be a key to more effective vaccination against chronic viruses, such as HIV-1, HCV, and others.

## Author Contributions

Dr. SM describes her invention of mAb 1F7 and subsequent experimental findings. Prof. MG describes his findings about HCV related to 1F7 idiotype expression. Dr. MP describes findings of 1F7 expression on neutralizing anti-HIV antibodies. Dr. HK has written an additional paragraph on general anti-idiotypic regulation.

## Conflict of Interest Statement

Matthew Parsons and Michael Grant serve on the scientific advisory board of Network Immunology Inc., a private company with an interest in developing vaccines against human immunoodeficiency virus. The authors declare that the research was conducted in the absence of any commercial or financial relationships that could be construed as a potential conflict of interest.
